# Ethics education for pediatric residents: a review of the literature

**Published:** 2015-04-20

**Authors:** Raywat Deonandan, Hafsa Khan

**Affiliations:** 1 Interdisciplinary School of Health Sciences, University of Ottawa, Ottawa, Ontario

## Abstract

**Background:**

Ethics education and research on medical residents is needed because, unlike medical students or experienced doctors, medical residents have to perform multiple roles simultaneously – student, teacher and clinician – thus exposing them to unique ethical stressors. In this paper we reviewed the literature concerning ethics education in postgraduate pediatrics training programs. Our goal was not to simply describe educational strategies and programs, but also to explore measurements and experiences of current practices to address gaps in ethics education during residency.

**Method:**

We conducted a structured literature review to explore the extent of ethics education in pediatric residency programs.

**Results:**

Twelve relevant studies were found. The studies suggest that existing training regimens are insufficient to meet the real life ethical challenges experienced in actual practice, particularly with respect to palliative care and the commission of clinical errors.

**Conclusions:**

The increasing diversity of culture and beliefs in the clinical workplace is also serving to complicate educational needs. An interdisciplinary approach, spread over the entirety of a physician’s training, is a proposed solution worthy of more attention.

## Introduction

Ethics education research related to medical residents is needed because, unlike medical students or experienced doctors, medical residents have to perform multiple roles simultaneously – student, teacher and clinician – thus exposing them to unique ethical stressors.^1^ According to McDougall, having to be a responsible clinician, learner, and a resource to patients and students all at once creates a unique set of ethical issues for medical residents.^1^ Additionally, for medical ethics education to significantly influence the “moral identities of physicians” at all levels of experience, such education has to pervade the daily experiences of medical residents.^2^ Thus, teaching medical ethics in a single, isolated course has been deemed insufficient.^2^ What is likely required is a more comprehensive ethics education program, folded into the larger residency experience.

Pediatric residencies are distinct from other specializations, in part due to what Baldwin et al. describe as the trial-and-error nature of the first few years of practice.^3^ In addition, children’s growth and development, often at variable rates, presents a challenge to ethics processes since the protocols for addressing the needs of a newborn differ from those of a teenager.^4^ There are certainly analogous challenges in other medical specialties. But when considered alongside the extreme time and emotional demands of the residency process, the complexities of a pediatric residency offer plentiful opportunities for ethical stumbles especially from the emotional weight of dealing with suffering children, and the difficulty in necessarily processing patient communication through a third party (the patient’s parent or guardian). The key to proper personal development in this role is not necessarily the avoidance of mistakes, but rather how those mistakes are handled by trainees.^3^

In recognition of the importance of ethics in all aspects of medical practice, ethics education in undergraduate medical school curricula has become essentially universal.^5^ Ethics and professionalism have also become integral parts of competency-based curricula for residents in nearly all specialties of medicine.^5^ And yet it is not clear to what extent postgraduate pediatric training routinely includes ethics education or skills training goes beyond a single course. Thus, in this paper we reviewed the literature concerning ethics education in postgraduate pediatrics training programs. Our goal was not to simply describe educational strategies and programs, but also to explore measurements and experiences of current practices to address gaps in ethics education during residency.

## Methods

We closely followed the methods of Helft *et al.*,^5^ who conducted a structured literature review concerning postgraduate ethics training in surgical residencies. Our goal was to review the published, peer-reviewed literature concerning ethics education during pediatrics residency and to elucidate areas of ethical education investment indicated by the literature.

We employed conjunctive searches of the terms “pediatrics,” “ethics education,” “residents,” and “pediatric residents” (including all accepted spelling usages). Next, we substituted our search using the search terms “clerkship,” “residency,” “medical ethics,” “pediatric residency programs,” and “postgraduate pediatric residency.”

Articles that did not pertain directly to the subject matter were excluded, namely those that: (1) primarily discussed professionalism (unless they specifically examined the ethical aspect of professionalism); (2) pertained to the ethics of how a school treats its residents; and (3) studied practicing pediatricians as opposed to pediatric residents. Papers dealing with residents’ ethical explorations in their personal lives were also excluded.

Our search was limited to Ovid Medline and the NLM PubMed, and to full-text papers published in the English language in 2005 or later. This cut-off date was chosen because it coincided with the last text publication date of the American Medical Association’s Code of Medical Ethics, and because a preliminary scan of the literature revealed a dearth of residency-based ethics papers published before 2005. The references lists of all the relevant papers were also searched, and the articles that fit our criteria were included in the final results.

## Results

Our initial search using our keywords resulted in 55 articles, which were reduced to 12 papers after the application of our three exclusion criteria. All 12 were empirical in nature, and were either U.S. or Canadian. One was a mixed methods study, one qualitative, one longitudinal, and the remaining nine were cross-sectional surveys. There were three articles discussing medical error disclosure by pediatric residents, two exploring ethical issues that physicians often encounter on a daily basis, one concerning the ethical knowledge and concerns of pediatric residents, and six articles assessing ethical education and training in pediatric residency programs. Of the latter six, two discussed ethics education in general, while the remaining four focused on palliative care.

We categorized the results into five thematic groups: studies about (1) medical error disclosure; (2) ethical issues faced in routine care; (3) exploring pediatric residents’ ethical knowledge and concerns; (4) evaluating ethics education in pediatric residency programs; and (5) palliative care training and education in pediatric residency.

### Studies assessing medical error disclosure

A study of 118 clinically active pediatric residents found that most had been involved in a medical error situation, and endorsed reporting medical errors to the hospital, but that a very small proportion (36%) actually reported serious errors to patients’ families.^6^

While most practitioners would accept that disclosure of an error is, in principle, an ethical necessity, evidence suggests that disclosure is affected by several contextual issues including the seriousness of the transgression, as less serious errors are more likely to be reported.^7^ Even reported errors suffer from incompleteness, with a minority of residents offering to apologize for a clinical error, or to offer full details explaining how the error had happened.^7^ Moreover, pediatric residents are much more likely to disclose medical errors when the errors are more obvious to the child patient’s parents than when they are not, indicating a deficit in ethical behaviour. The authors of one study state that “effective training strategies to help undergraduates, graduate-level trainees, and practicing physicians become more adept at error disclosure have not yet been established,” noting the obvious ethical deficit of failing to disclose errors in the face of clear evidence that patients prefer open disclosure. 7

A majority of residents agree that medical errors are one of the most serious problems in healthcare, that errors should be disclosed, and that disclosing errors would be difficult.^8^ Indeed, while 90% of residents were able to correctly identify the error in a given scenario, only 40% reported that they would disclose it.^8^ According to that study, very few would use the word “mistake,” or acknowledge that harm had been done. And most interesting is that only 40% of the residents said that they had received teaching on medical disclosure.

It seems that so-called “contextual factors” such as team environment, social relationships and individual seniority are commonly relied upon when making a decision about error disclosure. 8

### Studies exploring ethical issues faced in routine care

Our search revealed two studies^9^, ^10^ that investigated ethical issues faced by pediatric residents in routine care. A substantial proportion of residents have frequent ethical “confrontations” in the NICU, such as being compelled to resuscitate an infant according to parental wishes, but contrary to standard medical procedure. As per this example, difficulty with decisions regarding resuscitation varied with residents understanding of the underlying medicine. 9 It is therefore advisable to consider ethics principles ideally within the context of routine clinical care.

Six themes or factors arose within the two studies, each relating to the type of ethical challenge most likely to be encountered by a pediatric resident: (1) promoting the best interests of the child in resource-poor and complex social and home settings; (2) handling the therapeutic alliance with parents or caregivers; (3) protecting the privacy and confidentiality of patients; (4) finding a balance between the dual roles of healthcare provider and learner; (5) using professional authority appropriately (for example, to not encourage behaviours not immediately pertinent to clinical practice); 10 and (6) the role of cultural diversity, as residents in programs with more multicultural and pluralistic populations experienced more ethical confrontations than those with more culturally homogenous populations.^9^

With regard to cultural diversity (point 6 above), ethical confrontations are more likely to occur in situations where residents are from cultural backgrounds distinct from that of their patients. It can be argued that in ethical situations where residents have highly variable and competing values and beliefs, ethical confrontations are more likely to occur than in situations where residents’ beliefs are more similar to those of their colleagues and patients.^9^ The implication is that ethics training needs to be updated to reflect the real needs of residents operating in modern, diverse and typically urban settings.

### Studies exploring residents’ ethical concerns and knowledge

The authors of a 2005 study^11^ reported that 54% of residents working in pediatric intensive care units (PICUs) across the southern and eastern USA reported “At times, I have acted against my conscience in providing treatment to children in my care,” and that 15 times as many residents agreed with the statement, “Sometimes I feel we are saving children who should not be saved,” compared to those that agreed with the statement, “Sometimes I feel we give up on children too soon.”

The study also found that many residents held ethical views that differed considerably from published ethical recommendations, with the authors stating that they have “serious concerns of conscience about the provision of overly burdensome treatments for gravely ill children, which suggests that there are powerful barriers to establishing appropriate goals of care.” The authors concluded that there is a need in residency for more hospital-based ethics education and interdisciplinary discussions about complex and stressful pediatric end-of-life case studies. They reached this conclusion based upon their finding a need and desire for education around ethical guidelines for pediatric end-of-life decision-making, even among respondents whose knowledge of formal ethics theory is quite high.

### Studies that assessed ethics education in residency programs

The majority of articles (six) included in this study were evaluations of ethics education in pediatric residency programs. Two of those studies looked at ethics education in general, while the others focused on palliative care education specifically. Some general themes arose from the results of these studies. First, a large proportion of respondents rated their residency ethics education as poor or fair (45% in one study),^12^ despite an overwhelming tendency to report ethics education to be of paramount importance – comparable to the importance of basic clinical education.^13^ There is a prevailing perception that poor ethics education is related to low confidence in the ethics end-of-life care and decision-making, and in research ethics as well.^12^

Second, the modality of teaching ethics was perhaps insufficient to meet residents’ perceived needs. More than 80% of pediatricians found informal discussions with their colleagues and supervisors to have a moderate or major effect on their ethics education, whereas only 53% found formal teaching conferences to have the same effect.^12^ A real-life, evidence-based approach to ethics education seems to be most appealing to residents.^12^

Third, there was an acceptance that resource and scheduling limitations are real barriers to delivering proper ethics education, as is consensus on curriculum content. Attempting to develop a bioethics curriculum in isolation at individual residency programs only intensifies the challenges faced by programs in deciding what topics to teach and how to teach them.^13^ End-of-life and consent issues are consistently brought up as the most important ethical concerns but the relative importance of family issues, disclosure (of error, of bad news, and overall truthfulness), and issues pertaining to the medical industry, resource allocation, and research do not receive the same level of support across studies.

The University of Toronto experience is of particular interest, since the results of their study^13^ informed subsequent changes to the University’s ethics training regimen. The authors pointed out that a creative post-graduate bioethics education can create an opportunity for representatives from different disciplines and departments to share both their successful and unsuccessful experiences in teaching ethics, and that such discourse is necessary for developing a comprehensive, specialty-specific ethics curriculum. Indeed, multi- and interdisciplinary perspectives born of actual experiences consistently receive strong praise across studies as foundations for an ethics education program.

### Studies that assessed ethics in palliative care training and education

Four studies identified in our search explored the ethics of pediatric palliative care. This medical specialization is associated with larger ethical constructs, including the societal role of physician as arbiter between life and death and, as noted earlier,^11^ the subjective aspects of making end-of-life decisions, such as physician beliefs and biases. Palliative care for pediatric patients is particularly interesting within an ethics framework because end-of-life decisions, which can be considered among the medical decisions of greatest social and emotional gravity, are further complicated both by a need to morally and legally engage with a parent or guardian, and by the very fact of making such decisions for children, whose needs, care and societal role are traditionally viewed as distinct from those of adults.

Overall, residents feel that pediatricians should play an important role in providing palliative care, despite feeling that they have had minimal training, knowledge, experience, and comfort in nearly all areas of pediatric palliative care.^14^ Given that a great majority of residents can expect to care for a dying child at some point,^15^ educational needs must include pain management and control, and communication skills, especially with regard to discussing prognosis, delivering bad news, code status, and speaking to children about end-of-life care.^14^ A focus on symptom management and psychosocial support for patients, as well as some degree of spiritual support, are also indicated.^15^

While only a small minority (7%) of residents feel adequately prepared to deal with death and dying in their practice,^16^ a majority believe that the pediatric profession as a whole has not sufficiently explored issues related to death and dying, and that it does not effectively serve the needs of terminally ill children and their families.^15^ Indeed, residents report they had little or no training, experience, knowledge, comfort and competence in palliative care as a whole. 17 Palliative care principles should therefore be incorporated into pediatric residency education in order for them to be better implemented in practice, optimally through informal teaching and during rounds.^16^

One study suggests that the three most important subjects that residents thought should be taught in palliative care education are: discussing prognosis, delivering bad news, and controlling pain.^17^ Also worthy of consideration are supporting family spirituality and having emotional support for physicians. An additional recommendation is that palliative care be taught by observation, bedside teaching, and in multidisciplinary or interdisciplinary discussion groups.^17^ Current formal ethics training is typically accomplished through dedicated ethics grand rounds, which is widely considered not to be either multi- or interdisciplinary.^17^

## Conclusions

According to Helft *et al.*,^5^ there are two points of view in the literature about the primary goal of medical ethics education: to cultivate virtuous physicians, and to provide physicians-in-training with a set of skills to address ethical dilemmas they encounter in their practices in a well-reasoned way. Our findings suggest that the tendency is for researchers to mostly consider Helft’s second goal, that of seeking to prepare physicians for real-life situations.

Our findings indicate a disconnection between residents’ need for ethics training and the actual intensity and comprehensiveness of preparatory training for both palliative care and medical error issues. A grander inclusion of societal and contextual factors in education regarding guidance for decision-making appears to be indicated, as well as a need for better incorporation of diverse belief sets within the taught ethical frameworks.

One of the barriers to the uptake of these lessons is that many residency programs do not carry out formal evaluations of their ethics curricula.^18^ Often, ethics education is comprised of but a single class, while experiences indicate a need for continuing, experience- and context-based reaffirmations of ethical principles. Indeed, the main lesson extracted from this review is that the current medical education paradigm requires more hospital-based ethical training for pediatric residents, preferably of a more interdisciplinary nature.^11^, ^18^

A key finding was a demand for more “real-life” context cases. Such scenarios would be representative both of potential and actual clinical experiences, but also of clinic settings outside of hospital-based care, such as community outreach or the contextualization of social determinants of health within immediate clinical practice.

A common message is repeated in almost all of the reviewed studies: ethics education needs to be improved to meet the unique situational needs of pediatric residents. Of particular note is the University of Toronto model^13^ which seeks to address issues of comprehensiveness and varied stakeholder perceptions; it can be emulated in diverse, mostly urban populations. Longitudinal studies are needed to determine the extent to which all educational models result in consistent and effective ethical behaviors.

The number of studies conducted on the ethics education needs and provisions for pediatric residents is sparse. In this study, we have focused on qualitative aspects of existing training experiences, as expressed via published studies. However, it is likely that many training regimens have not been expressed via the peer-reviewed literature, and thus would not have appeared in our search results. The shared message of the reviewed studies, though, is that existing training regimens are insufficient to meet the real life ethical challenges experienced in actual practice, particularly with respect to palliative care and the commission of clinical errors. The increasing diversity of culture and beliefs is also serving to complicate educational needs. An interdisciplinary approach, spread over the entirety of a physician’s training, is a proposed solution. Such an approach would consider that professionals from various disciplines hold different perspectives on a range of inherently complex issues in the provision of care to gravely ill children.^11^

Future directions for research on this subject should include the development of specific methods for incorporating ethics education into pediatrics residency training, both informally and formally, within institutions’ resource limitations. Error reporting systems should be improved, including training in how best to disclose clinical errors, to both increase patient trust and prevent future medical errors.^6^ Additionally, more in-depth reviews of existing ethics training programs, or programs currently in development, would be useful, especially with respect to determining their impacts on residents’ behaviours and perceptions of preparedness.
